# Optimizing Impella support level with intracardiac echocardiography during ventricular fibrillation storm ablation under veno-arterial extracorporeal membrane oxygenation

**DOI:** 10.1007/s10396-026-01618-8

**Published:** 2026-02-17

**Authors:** Yuhei Kasai, Takayuki Kitai, Junji Morita, Ryo Horita, Ryo Otake, Jungo Kasai, Daisuke Hachinohe

**Affiliations:** 1 Department of Cardiology, Sapporo Cardiovascular Clinic, North 49, East 16, 8-1 Higashi Ward, Sapporo, Hokkaido 007-0849 Japan; 2 Toyota Technological Institute at Chicago, Chicago, IL USA

A 61-year-old man was admitted to our hospital with heart failure with reduced ejection fraction, complicated by subacute myocardial infarction (onset 4 days previously). On hospital day 10, he developed ventricular fibrillation (VF) storm, necessitating emergent initiation of veno-arterial extracorporeal membrane oxygenation (VA-ECMO) and Impella CP support via right and left femoral access, respectively. Subsequently, coronary angiography was performed, followed by percutaneous coronary intervention for the right coronary artery (Fig. 1a). On hospital day 13, the Impella was upgraded from CP to 5.5 for heart failure management. Despite sedation, analgesia, and antiarrhythmic therapy with amiodarone and landiolol, recurrent VF storm occurred on day 15 and proved refractory, necessitating emergent catheter ablation on day 16 (Fig. 1b).

**Fig. 1:**
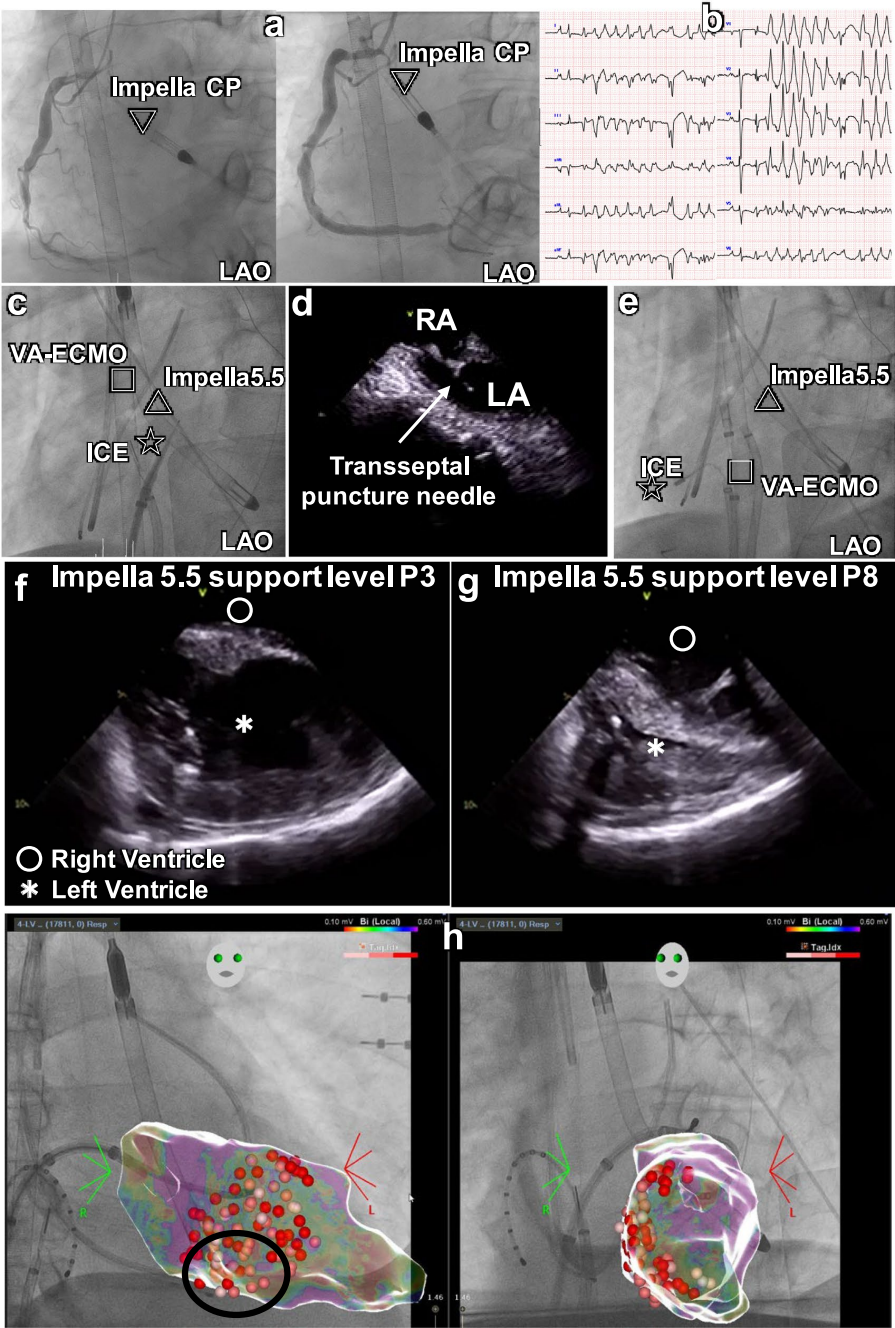
**a** Right coronary angiograms showing the pre-intervention state (left panel; black arrow indicates the occluded segment) and post-intervention result (right panel). **b** Twelve-lead electrocardiogram at the onset of VF. **c**, **d** Fluoroscopic (**c**) and ICE (**d**) images obtained during transseptal puncture. **e** Fluoroscopic view of the ICE catheter advanced into the right ventricle. **f**, **g** ICE images of the LV during Impella P3 (**f**) and P8 (**g**) support. P3: mean arterial pressure 70 mmHg, ECMO flow 4.2 L/min, pulsatility index 0.43. P8: mean arterial pressure 73 mmHg, ECMO flow 3.0 L/min, pulsatility index 0.19. **h** Three-dimensional electroanatomical mapping image during ablation (Purkinje de-networking). At the black circle (indicated site), catheter stimulation reproducibly induced VF. *LAO* left anterior oblique, *VAECMO* veno-arterial extracorporeal membrane oxygenation, *ICE* intracardiac echocardiography, *RA* right atrium, *LA* left atrium

A steerable sheath (Biosense Webster, Diamond Bar, CA) was introduced via the left femoral vein, followed by transseptal puncture under intracardiac echocardiography (ICE) guidance (SoundStar™; Biosense Webster) (Fig. 1c and d). During the ablation procedure, an ICE catheter was advanced into the right ventricle to evaluate the optimal Impella setting (Fig. 1e). When the P-level was increased from 3 to 8 (Fig. 1f and g), ICE showed near-complete collapse of the left ventricular (LV) cavity, which was anticipated to hinder catheter manipulation within the LV (Fig. 1g) (Supplemental Video 1). Reducing the P-level to P3 eliminated the electromagnetic interference at P8, with sufficient LV cavity preservation (Supplemental Videos 1 and 2). LV electroanatomical mapping was performed using a high-density mapping catheter (Biosense Webster). Subsequently, Purkinje de-networking and extensive ablation were performed in the left posterior fascicle region, where positioning of the mapping catheter induced VF (Fig. 1h). The procedure was completed without complications. VA-ECMO was discontinued on day 19, and the Impella was removed on day 22.

VF occurring after myocardial infarction often involves the Purkinje network [1]. Purkinje de-networking is an effective strategy for a drug-refractory VF storm. Continuous ICE monitoring allowed us to maintain the lowest Impella support level necessary to preserve the hemodynamic stability and mapping quality while minimizing interference between the catheters and Impella [2]. This is the first case report of catheter ablation for drug-refractory VF storm after myocardial infarction in a patient with VA-ECMO and Impella support, where the optimal Impella support level was determined using ICE without additional imaging modalities. Transesophageal echocardiography can be used as an alternative, but ICE offers advantages such as direct control of the imaging plane and continuous hemodynamic monitoring during the procedure.

## Supplementary Information

Below is the link to the electronic supplementary material.

Supplementary file1 (MP4 24557 KB) ICE recordings demonstrating changes in left ventricular (LV) cavity size as the P-level is adjusted from P3 to P8 and then returned to P3 (shown at 4× normal speed).

Supplementary file2 (MP4 17758 KB) With the Impella support level at P8, the ablation catheter appears unstable on the three-dimensional mapping system, despite remaining stationary, due to electromagnetic interference.

## Data Availability

No additional datasets were generated or analyzed for this case report.
